# Microbial Diversity and Composition in Six Different Gastrointestinal Sites among Participants Undergoing Upper Gastrointestinal Endoscopy in Henan, China

**DOI:** 10.1128/spectrum.00645-21

**Published:** 2022-04-25

**Authors:** Minjuan Li, Dantong Shao, Jiachen Zhou, Jianhua Gu, Junjie Qin, Xinqing Li, Changqing Hao, Wenqiang Wei

**Affiliations:** a National Central Cancer Registry, National Cancer Center/National Clinical Research Center for Cancer/Cancer Hospital, Chinese Academy of Medical Sciences and Peking Union Medical College, Beijing, China; b Department of Epidemiology and Biostatistics, School of Public Health, Xi’an Jiaotong University Health Science Center, Xi’an, China; c Promegene Institute, Shenzhen, China; d Department of Endoscopy, Cancer Institute/Hospital of Linzhou, Linzhou, China; Huazhong University of Science and Technology

**Keywords:** gastric cardia, gastric juice, gastric pH, gastrointestinal tract, *Helicobacter pylori*, microbiota

## Abstract

The objective of this study was to describe and compare the dynamic microbiota characteristics in the gastrointestinal (GI) tract in Chinese participants via high-throughput sequencing techniques. The study collected saliva, esophageal swab, cardia biopsy, noncardia biopsy, gastric juice, and fecal specimens from 40 participants who underwent upper GI tract cancer screening in Linzhou (Henan, China) in August 2019. The V4 region of 16S rRNA genes was amplified and sequenced using the Illumina MiniSeq platform. The observed amplicon sequence variants (ASVs) gradually decreased from saliva to esophageal swab, cardia biopsy, noncardia biopsy, and gastric juice specimens and then increased from gastric juice to fecal specimens (*P *< 0.05). Each GI site had its own microbial characteristics that overlapped those of adjacent sites. Characteristic genera for each site were as follows: Neisseria and Prevotella in saliva, Streptococcus and Haemophilus in the esophagus, Helicobacter in the noncardia, Pseudomonas in gastric juice, Faecalibacterium, Roseburia, and Blautia in feces, and Weissella in the cardia. Helicobacter pylori-positive participants had decreased observed ASVs (cardia, *P *< 0.01; noncardia, *P *< 0.001) and Shannon index values (cardia, *P* < 0.001; noncardia, *P *< 0.001) compared with H. pylori-negative participants both in cardia and noncardia specimens. H. pylori infection played a more important role in the microbial composition of noncardia than of cardia specimens. In gastric juice, the gastric pH and H. pylori infection had similar additive effects on the microbial diversity and composition. These results show that each GI site has its own microbial characteristics that overlap those of adjacent sites and that differences and commonalities between and within microbial compositions coexist, providing essential foundations for the continuing exploration of disease-associated microbiota.

**IMPORTANCE** Upper gastrointestinal (UGI) tract cancer is one of the most common cancers worldwide, while limited attention has been paid to the UGI microbiota. Microbial biomarkers, such as Fusobacteria nucleatum and Helicobacter pylori, bring new ideas for early detection of UGI tract cancer, which may be a highly feasible method to reduce its disease burden. This study revealed that each gastrointestinal site had its own microbial characteristics that overlapped those of adjacent sites. There were significant differences between the microbial compositions of the UGI sites and feces. Helicobacter pylori played a more significant role in the microbial composition of the noncardia stomach than in that of the cardia. Gastric pH and Helicobacter pylori had similar additive effects on the microbial diversity of gastric juice. These findings played a key role in delineating the microbiology spectrum of the gastrointestinal tract and provided baseline information for future microbial exploration covering etiology, primary screening, treatment, outcome, and health care products.

## INTRODUCTION

Gastrointestinal (GI) microbiota refers to the resident and transient bacteria, viruses, fungi, protozoa, and archaea in the human GI tract and can be considered an organ that coevolved with the host to maintain physiological homeostasis. Microbial homeostasis is vital to the physiological balance of the host, playing key roles in development, acquisition of nutrients, immunity, and multiple biological processes (1–3). Once the microbiota becomes unbalanced, the microenvironment may change, leading to disease (4). To date, scientific clues about the relationship between dysbacteriosis and diseases, including diabetes, obesity, cancer, and mental illness, are emerging (5–8). The development of microbial studies is gradually uncovering the significance of microbiota in the early detection and early diagnosis of disease by microbial biomarkers like Fusobacteria nucleatum (9, 10) and Helicobacter pylori. Additionally, increasing evidence indicates that targeted reconstitution of the gut microbiota, such as by fecal microbiota transplantation (FMT), is an ideal therapeutic strategy against gastrointestinal disorders (11). Apparently, the GI microbiota is closely related to health and disease.

Currently, there is more emphasis on single GI sites or multiple specimens from one site, such as feces (12), saliva, the tongue dorsum, and supragingival plaque from the oral cavity (13), or on paired esophageal biopsy and swab specimens from the esophagus (14). In multisite microbial studies (15, 16), there is limited evidence regarding the upper gastrointestinal (UGI) sites, especially the esophagus and cardia, and meanwhile, there is a heavy global burden of UGI cancer (17). To decrease the morbidity and mortality of UGI cancer, early detection is an effective method (18). However, it is still unclear how feasible it may be to replace difficult-to-collect esophageal or gastric specimens with easy-to-collect saliva or fecal specimens for early detection of UGI cancer and precancerous lesions by detection of microbial biomarkers during primary screening. Additionally, H. pylori infection plays an important role in the progression of gastric cancer. The alpha diversity in the stomach is lower in patients with H. pylori-positive status than in patients with H. pylori-negative status (19, 20). H. pylori infection has no significant effect on the microbiota diversity and composition in gastric juice (20). Nevertheless, it is still unclear whether H. pylori infection and gastric pH have an influence on the microbial characteristics in other sites. Moreover, individual differences are a nonnegligible barrier to microbial studies, since they influence the required intensive study, data mining and integration, and translational application.

Considering the status of microbial research described above, we conducted a gastrointestinal microbiota study in China to describe and compare the microbial diversities, microbial compositions, and dynamic changes of bacterial populations existing in the oral cavity, esophagus, cardia, noncardia stomach, gastric juice, and feces. This is a crucial step to map the microbial composition, which is of great value to demonstrate and underpin the bacterium-associated diseases affecting human health.

## RESULTS

### Baseline information for participants.

A total of 40 participants were enrolled in this study, including 22 healthy participants and 18 participants with esophagitis. Half of the participants in both the healthy and the esophagitis group had H. pylori infection. All participants were negative for fecal occult blood testing. Baseline information is summarized in Table 1.

**TABLE 1:** Demographic information of enrolled participants

Epidemiological factor	No. (%) of participants or value as indicated in the following group^*a*^:		
	Healthy (*n* = 22)	Esophagitis (*n* = 18)	Total (*n* = 40)
Male	9 (40.91)	9 (50.00)	18 (45.00)
≥56 yrs of age	7 (31.82)	11 (61.11)	18 (45.00)
Median age ± SD (yr)	53 ± 7.49	58 ± 6.72	54 ± 7.20
Married	20 (90.91)	18 (100.00)	38 (95.00)
Agriculture and farming	10 (45.45)	14 (77.78)	24 (60.00)
Primary school or below	11 (50.00)	6 (33.33)	17 (42.50)
Smoker	2 (9.09)	3 (16.67)	5 (12.50)
Never or rarely drinks tea	21 (95.45)	18 (100.00)	39 (97.50)
Eats rice at least 1 to 3 days/wk	18 (81.82)	14 (77.78)	32 (80.00)
Eats pasta every day	16 (72.73)	8 (44.44)	24 (60.00)
Eats harder staple food	8 (36.36)	2 (11.11)	10 (25.00)
Eats livestock meat at least 1 to 3 days/mo	11 (50.00)	4 (22.22)	15 (37.50)
Eats eggs at least 1 to 3 days/mo	11 (50.00)	7 (38.89)	18 (45.00)
Never or rarely drinks milk	20 (90.91)	16 (88.89)	36 (90.00)
Eats vegetables every day	22 (100.00)	17 (94.44)	39 (97.50)
Eats fruits at least 1 to 3 days/mo	15 (68.18)	6 (33.33)	21 (52.50)
Never or rarely eats spicy food	21 (95.45)	16 (88.89)	37 (92.50)
Never or rarely eats hot food	22 (100.00)	18 (100.00)	40 (100.00)
Eats faster	6 (27.27)	4 (22.22)	10 (25.00)
Eats leftovers at least once/wk	9 (40.91)	12 (66.67)	21 (52.50)
Has lost 1 to 3 teeth	8 (36.36)	7 (38.89)	15 (37.50)
H. pylori infection	11 (50.00)	9 (50.00)	20 (50.00)
pH of gastric juice of <2	16 (72.73)	12 (66.67)	28 (70.00)
Median pH ± SD	1 ± 1.47	1 ± 1.70	1 ± 1.57
Family history of cancer	19 (86.36)	11 (61.11)	30 (75.00)

a None of the participants drank alcohol. All participants ate regular meals, three meals a day. There was no statistical difference in each factor between the healthy group and the group with esophagitis.

### Microbial characteristics in six GI sites.

**FIG 1:**
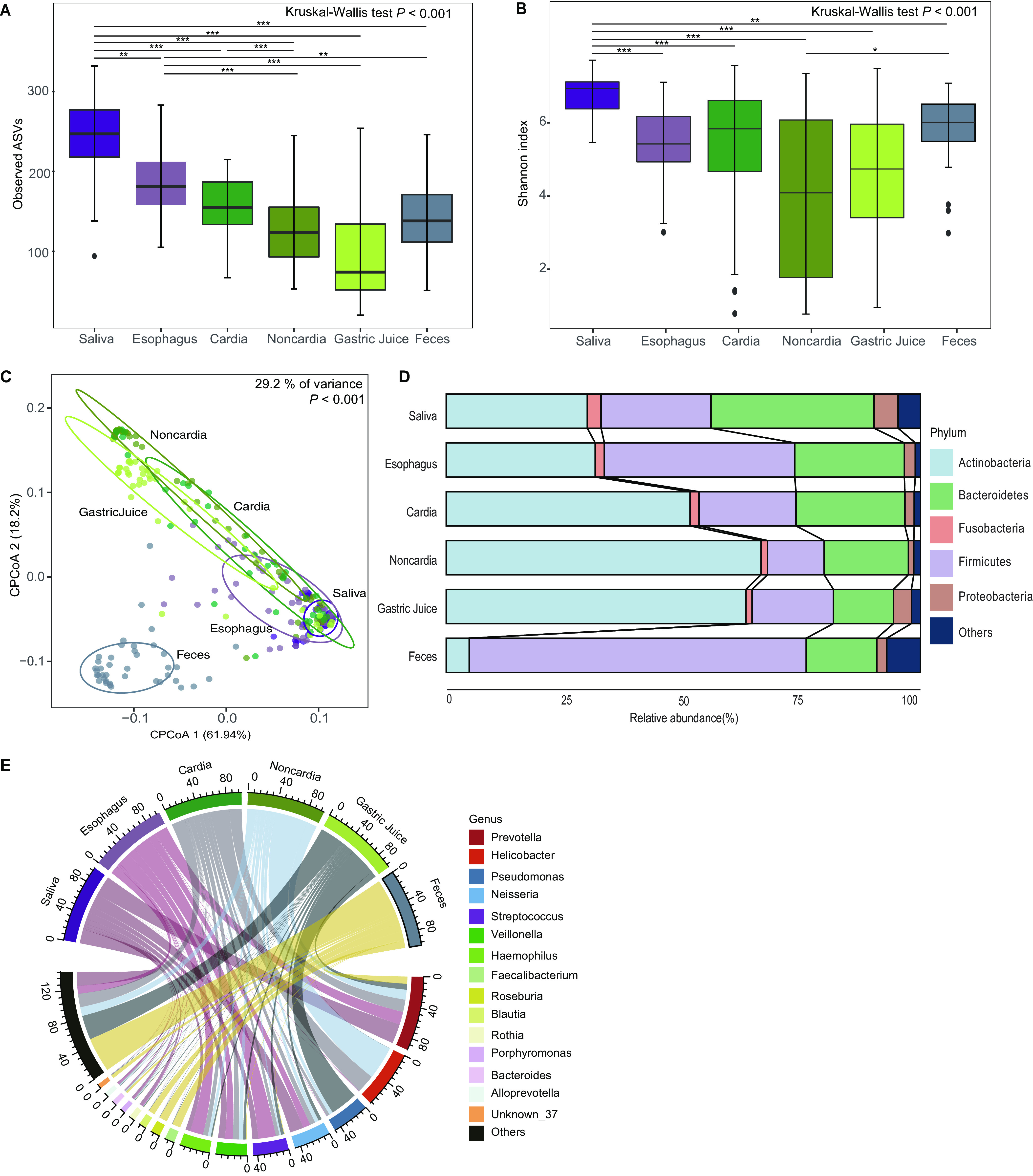
Microbial diversity and microbial composition in different GI sites. (A) Observed ASVs in different GI sites. Horizontal lines link groups that are statistically different by multiple testing after the Kruskal-Wallis test. ** and *** above the lines indicate *P* values after Bonferroni correction of less than 0.01 and 0.001, respectively. (B) Shannon index values in different GI sites. Horizontal lines link groups that are statistically different by multiple testing after the Kruskal-Wallis test. *, **, and *** above the lines indicate *P* values after Bonferroni correction of less than 0.05, 0.01, and 0.001, respectively. (C) CPCoA based on weighted UniFrac distance in different GI sites. (D) Microbial compositions including the top 5 phyla in different GI sites. (E) Microbial compositions including the top 15 genera in different GI sites. Top, microbial composition of the top 15 genera in different GI sites; bottom, source composition indicated the composition of different GI sites for each genus.

The observed amplicon sequence variants (ASVs) (Fig. 1A) gradually decreased from saliva to esophageal swab, cardia biopsy, noncardia biopsy, and gastric juice specimens and then increased from gastric juice to fecal specimens, which was not obvious in the Shannon index values (Fig. 1B). Taking into consideration multiple-comparison results both in observed ASVs (Fig. 1A) and Shannon index values (Fig. 1B), the alpha diversity was predominantly higher in saliva specimens than in other sites. For beta diversity, the constrained principal coordinate analysis (CPCoA) results presented in Fig. 1C showed there were differences in microbial composition among different GI sites and the microbial composition of feces was distinctly different from those of UGI sites. To be precise, around 0.13%, 0.12%, 0.16%, 0.07%, and 0.21% of the fecal microbiota come from saliva, esophageal swab, cardia biopsy, noncardia biopsy and gastric juice respectively (Fig. S1 in the supplemental material). The phyla *Actinobacteria*, *Bacteroidetes*, *Fusobacteria*, *Firmicutes*, and *Proteobacteria* were dominant in all GI sites. For each phylum, the dynamic changes in relative abundance are shown in Fig. 1D. Only *Fusobacteria* decreased from saliva to esophageal swab, cardia biopsy, noncardia biopsy, gastric juice, and fecal specimens. The relative abundances were 2.90%, 1.93%, 1.87%, 1.38%, 1.32%, and less than 0.01%, respectively. At the genus level, Alloprevotella, Bacteroides, Blautia, Faecalibacterium, Haemophilus, Helicobacter, Neisseria, Porphyromonas, Prevotella, Pseudomonas, Roseburia, Rothia, Streptococcus, and Veillonella were the predominant genera in all GI sites (Fig. 1E). Different GI sites had distinctive microbial compositions due to having different proportions of these genera (Table S1).

**FIG 2:**
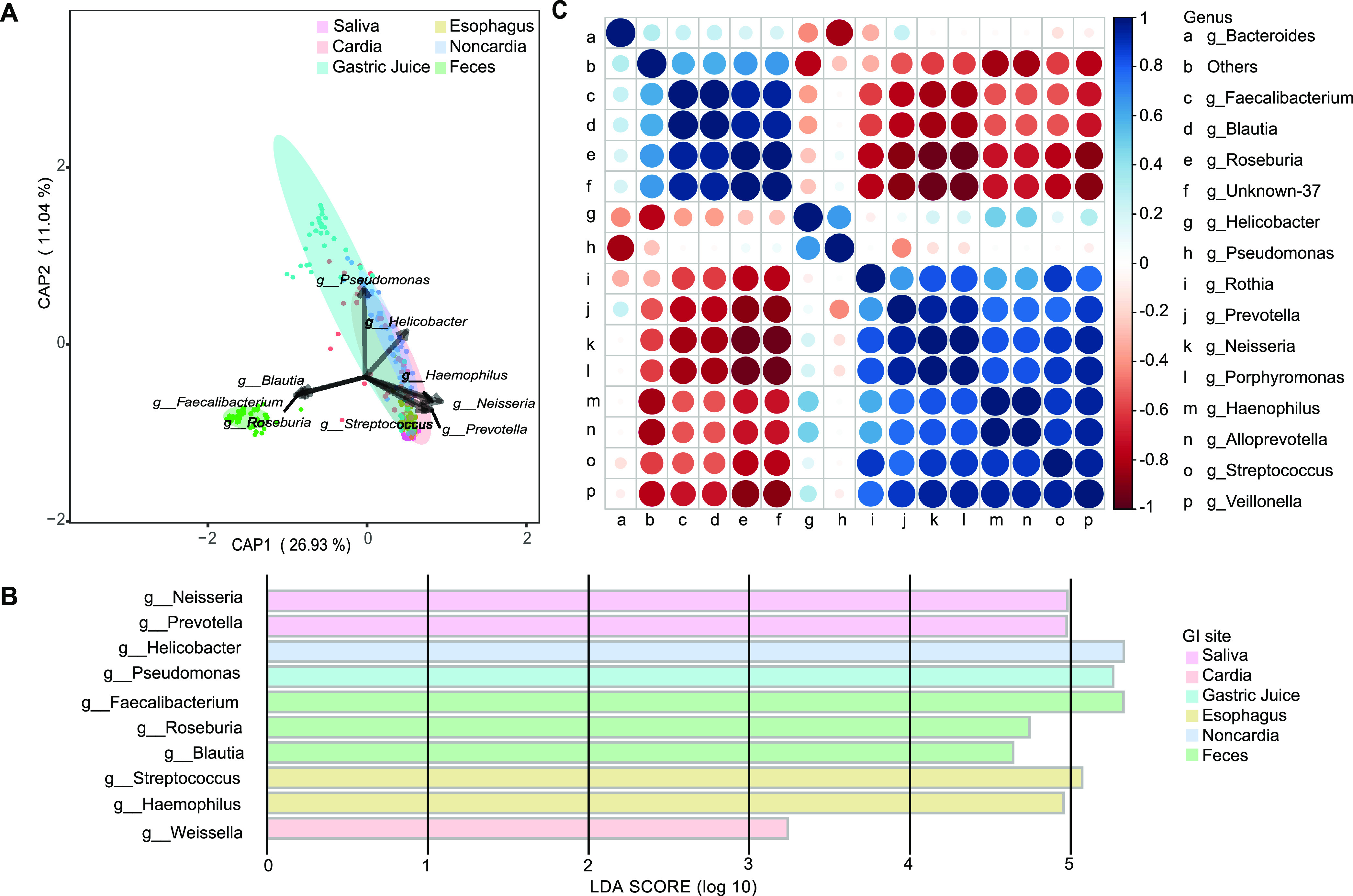
Characteristic genera in different GI sites. (A) db-RDA plot of characteristic genera in each GI site. (B) LEfSe results for characteristic genera in different GI sites. (C) Correlation plot of top 15 genera in six GI sites. CAP, constrained analysis of proximities.

Combining the results from distance-based redundancy analysis (db-RDA) (Fig. 2A) and linear discriminant analysis effect size (LEfSe) (Fig. 2B, Fig. S2), we found the characteristic genera among the top 15 genera for each GI site to be as follows: Neisseria and Prevotella in saliva, Streptococcus and Haemophilus in the esophagus, Helicobacter in the noncardia stomach, Pseudomonas in gastric juice, Faecalibacterium, Roseburia, and Blautia in feces, and none in the cardia. For the GI sites with two or more characteristic genera, correlation analysis was performed. Highly positive and statistically different correlations were observed in the characteristic genera of saliva (Neisseria and Prevotella, coefficient = 0.64), the esophagus (Streptococcus and Haemophilus, coefficient = 0.75), and feces (Faecalibacterium and Roseburia, coefficient = 0.78; Faecalibacterium and Blautia, coefficient = 0.76; and Roseburia and Blautia, coefficient = 0.79) (Fig. 2C). Helicobacter was strongly positively correlated with Pseudomonas, and these two genera were negatively correlated with Bacteroides, which was a characteristic genus of feces. Expanding the limitation from the top 15 genera to all genera, the most representative genus of the cardia was Weissella, as analyzed by LEfSe (Fig. 2B, Fig. S2). Additionally, characteristic metabolism pathways for each specimen type are summarized in Table S2, with a comparison to those of the saliva group.

### H. pylori infection affected the microbial characteristics of cardia and noncardia biopsy specimens.

**FIG 3:**
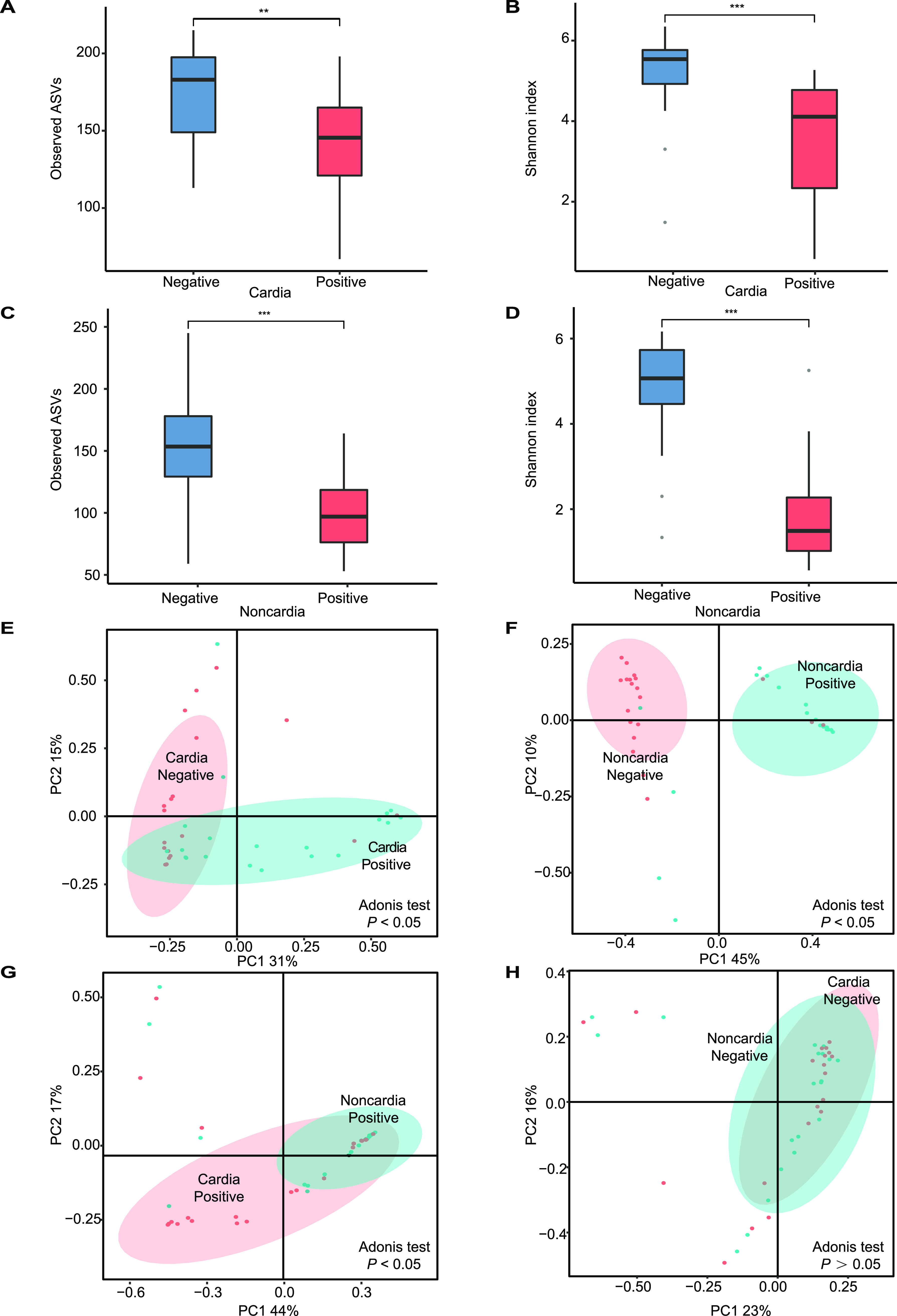
Effects of H. pylori infection on microbial diversity in cardia and noncardia biopsy specimens. (A) Observed ASVs between H. pylori-positive and H. pylori-negative cardia biopsy specimens. (B) Shannon index values between H. pylori-positive and H. pylori-negative cardia biopsy specimens. (C) Observed ASVs between H. pylori-positive and H. pylori-negative noncardia biopsy specimens. (D) Shannon index values between H. pylori-positive and H. pylori-negative noncardia biopsy specimens. *, **, and *** indicate *P* values of less than 0.05, 0.01, and 0.001, respectively. (E) PCoA of H. pylori infection in cardia biopsy specimens. (F) PCoA of H. pylori infection in noncardia biopsy specimens. (G) PCoA of H. pylori-positive status between cardia and noncardia biopsy specimens. (H) PCoA of H. pylori-negative status between cardia and noncardia biopsy specimens.

The observed ASVs and Shannon index values were lower in H. pylori-positive participants than in H. pylori-negative participants both in cardia (Fig. 3A and B) and noncardia (Fig. 3C and D) specimens. Both in H. pylori-positive (observed ASVs, *P *< 0.01; Shannon index, *P *< 0.01) and H. pylori-negative (observed ASVs, *P *= 0.05; Shannon index, *P *> 0.05) participants, the observed ASVs and Shannon index values were higher in cardia than in noncardia specimens. For beta diversity, the microbial community was significantly affected by H. pylori infection status, especially for noncardia specimens (Fig. 3E and F). Furthermore, in comparison to the results for cardia and noncardia specimens from H. pylori-positive participants (Fig. 3G), a large-scale overlap was shown between cardia and noncardia specimens of participants without H. pylori infection (Fig. 3H).

The top 5 phyla in cardia and noncardia biopsy specimens were *Proteobacteria*, *Bacteroidetes*, *Firmicutes*, *Actinobacteria*, and *Fusobacteria*. The relative abundances of all phyla except *Proteobacteria* were significantly reduced in noncardia biopsy specimens from the H. pylori-positive group compared with their abundances in cardia biopsy specimens (Table 2). The top 15 genera in cardia biopsy specimens were Helicobacter, Prevotella, Veillonella, Neisseria, Haemophilus, Streptococcus, Pseudomonas, Brevundimonas, Alloprevotella, Fusobacterium, Porphyromonas, Bacteroides, Selenomonas, Stenotrophomonas, and Delftia. However, the genus Stenotrophomonas in cardia biopsy specimens was replaced by Megasphaera in the top 15 genera in noncardia biopsy specimens. Stenotrophomonas and Megasphaera were statistically reduced in the H. pylori-positive group compared to their levels in the H. pylori-negative group both in cardia and noncardia biopsy specimens.

**TABLE 2:** Comparison of dominant phyla and genera in cardia and noncardia biopsy specimens

Taxon	Avg relative abundance (%) in indicated biopsy specimen type from participants with indicated H. pylori status				*P* value for indicated pairwise comparison^*a*^			
	Cardia		Noncardia					
	Positive	Negative	Positive	Negative	PC/NC	PN/NN	PC/PN	NC/NN
Phyla								
* Proteobacteria*	60.56	42.25	86.39	46.35	**	***	***	0.53
* Bacteroidetes*	15.35	30.54	6.79	28.73	***	***	**	0.74
* Firmicutes*	20.02	20.91	5.56	18.30	0.44	***	***	0.24
* Actinobacteria*	1.49	2.48	0.45	1.82	*	***	**	0.56
* Fusobacteria*	1.78	1.95	0.40	2.36	0.38	***	*	0.41

Genera								
* Helicobacter*	42.56	10.17	79.99	13.91	***	***	***	0.50
* Prevotella*	11.67	22.77	5.71	22.30	**	**	*	0.80
* Veillonella*	9.22	7.90	2.83	9.65	0.78	**	0.05	0.21
* Neisseria*	7.64	7.19	2.55	8.69	0.51	***	0.20	0.82
* * Haemophilus	5.07	8.72	1.28	9.23	0.11	***	*	0.58
* * Streptococcus	6.02	4.71	1.49	7.09	0.97	*	**	0.19
* * Pseudomonas	1.78	6.77	1.35	3.10	*	*	0.84	0.72
* Brevundimonas*	0.81	2.33	0.23	1.96	0.19	*	0.52	0.97
* Alloprevotella*	0.52	2.25	0.27	1.71	**	***	*	0.58
* Fusobacterium*	1.09	1.36	0.22	1.47	0.34	***	*	0.35
* Porphyromonas*	0.91	1.48	0.19	1.41	0.40	***	*	0.86
* Bacteroides*	0.93	1.36	0.23	0.93	0.34	*	***	0.25
* Selenomonas*	0.39	1.13	0.10	0.88	*	**	0.09	0.21
* Delftia*	0.46	0.83	0.17	0.73	0.20	**	0.11	0.60

a PC, H. pylori-positive cardia group; PN, H. pylori-positive noncardia group; NC, H. pylori-negative cardia group; NN, H. pylori-negative noncardia group; *, *P* < 0.05; **, *P* < 0.01; ***, *P* < 0.001.

For the above-described dominant genera, Table 2 shows the relative abundances in cardia and noncardia biopsy specimens in H. pylori-positive and H. pylori-negative participants. H. pylori infection statistically increased the relative abundances of Helicobacter both in cardia (increased 3.18 times) and noncardia (increased 4.75 times) biopsy specimens. Apart from Helicobacter, the relative abundances of all other genera were statistically decreased in noncardia biopsy specimens from H. pylori-positive participants. The relative abundances of Pseudomonas and Selenomonas were significantly lower in the H. pylori-positive group than in the H. pylori-negative group in both cardia and noncardia biopsy specimens and were similar between cardia and noncardia biopsy specimens in both the H. pylori-positive and the H. pylori-negative group. H. pylori infection expressly reduced the relative abundances of Prevotella (74.40% reduction) and Alloprevotella (84.59% reduction) in noncardia biopsy specimens. In addition, besides its effects on the microbial composition, H. pylori infection had an influence on the human disease (cancer and cardiovascular disease) pathways in noncardia biopsy specimens (Table S3).

### H. pylori infection and pH value had an influence on the microbial characteristics of gastric juice.

**FIG 4:**
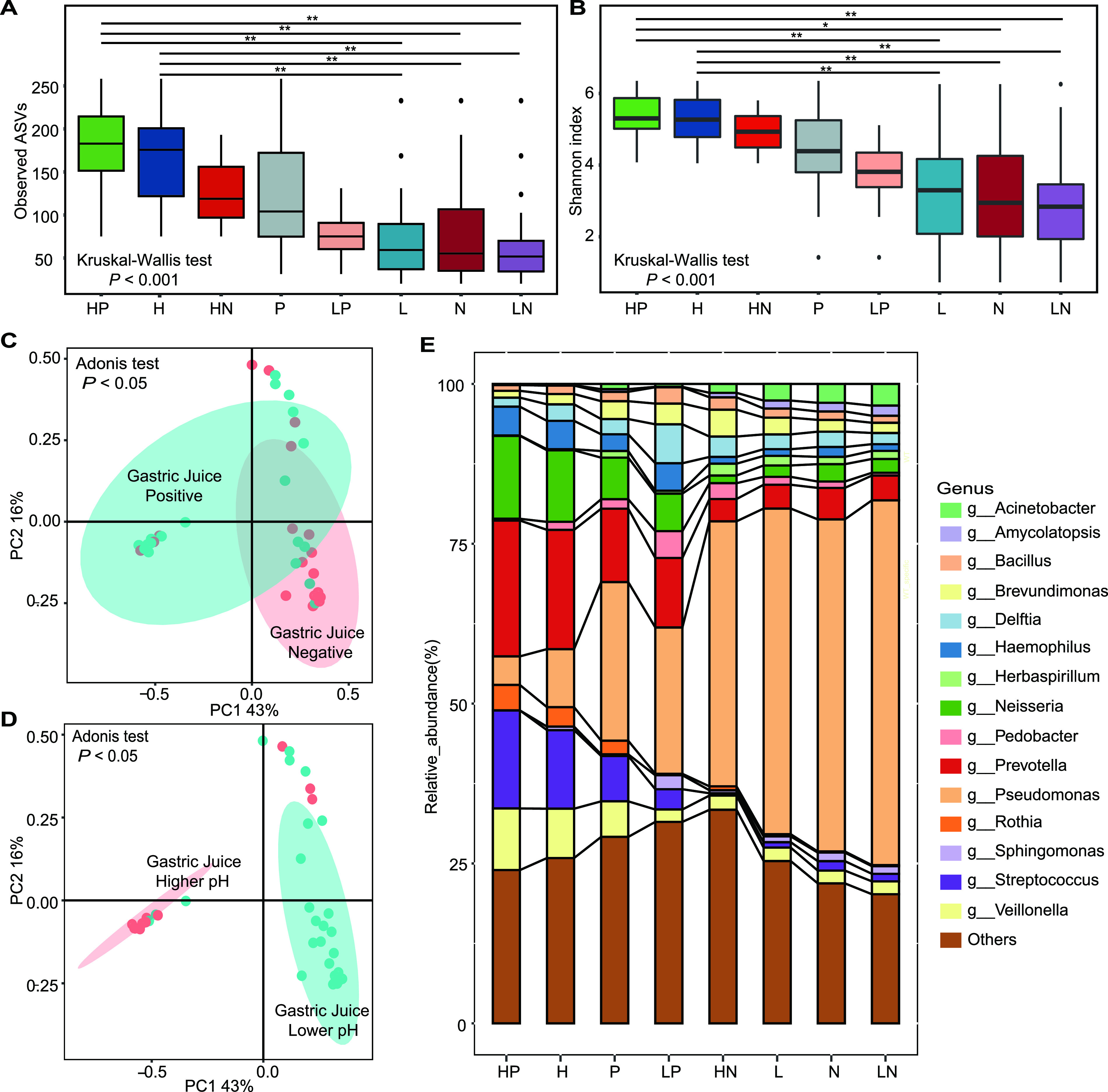
Microbial diversity and microbial composition in gastric juice. Definitions of groups are as follows: HP group, higher pH and H. pylori positive; H group, higher pH; HN group, higher pH and H. pylori negative; P group, H. pylori positive; LP group, lower pH and H. pylori positive; L group, lower pH; N group, H. pylori negative; LN group, lower pH and H. pylori negative. (A) Observed ASVs in gastric juice. Horizonal lines link groups that are statistically different by multiple testing after the Kruskal-Wallis test. ** above the lines indicates *P* values after Bonferroni correction of less than 0.01. (B) Shannon index values in gastric juice. Horizontal lines link groups that are statistically different by multiple testing after the Kruskal-Wallis test. * and ** above the lines indicate *P* values after Bonferroni correction of less than 0.05 and 0.01, respectively. (C) PCoA of H. pylori infection status in gastric juice. (D) PCoA of pH values in gastric juice. (E) Microbial composition including the top 15 genera in gastric juice.

When both H. pylori infection and gastric pH were considered, the observed ASVs (Fig. 4A) and Shannon index (Fig. 4B) decreased from the higher-pH and H. pylori-positive (HP) group to the higher-pH (H) group, the higher-pH and H. pylori-negative (HN) group, the H. pylori-positive (P) group, the lower-pH and H. pylori-positive (LP) group, the lower-pH (L) group, the H. pylori-negative (N) group, and last, the lower-pH and H. pylori-negative (LN) group. The alpha diversity of the HP group was surprisingly higher than those of the other groups, and there was an additional effect of H. pylori infection and gastric pH on the alpha diversity of gastric juice. However, there was no statistical difference between the alpha diversities of the HP group (observed ASVs = 177.33, Shannon index = 5.23) and the H group (observed ASVs = 164.75, Shannon index = 5.14). For beta diversity, both H. pylori infection (Fig. 4C) and pH value (Fig. 4D) influenced the microbial community in gastric juice, although pH value played a more prominent role.

Regarding microbial composition, the phyla *Proteobacteria* (*P *< 0.001), *Firmicutes* (*P *< 0.01), *Bacteroidetes* (*P *< 0.01), *Actinobacteria* (*P *< 0.05), and *Fusobacteria* (*P *< 0.01) were dominant in gastric juice. Among them, only the relative abundances of *Proteobacteria*, increased gradually from the HP group to the H group, the P group, the LP group, the HN group, the L group, the N group, and finally, the LN group. At the genus level, the top 15 genera were Prevotella, Streptococcus, Neisseria, Veillonella, Haemophilus, Pseudomonas, Rothia, Delftia, Brevundimonas, Bacillus, Pedobacter, Acinetobacter, Herbaspirillum, Amycolatopsis, and Sphingomonas (Fig. 4E). The genera Pseudomonas, Acinetobacter, and Rothia were influenced by H. pylori infection and pH value (Table S4). Additionally, the LEfSe results showed that Rothia was the characteristic genus in the P group, while Pseudomonas and Acinetobacter were the characteristic genera in the N group. The relative abundances of Prevotella, Neisseria, Streptococcus, Veillonella, Haemophilus, Herbaspirillum, and Amycolatopsis were significantly different between the H group and the L group. Pseudomonas, Acinetobacter, Herbaspirillum, and Amycolatopsis were the characteristic genera in the L group by LEfSe. Taking all comparisons based on various grouping methods into consideration, only Pseudomonas, Acinetobacter, and Rothia were significantly different between groups (Table S4).

### Microbial characteristics between and within participants.

**FIG 5:**
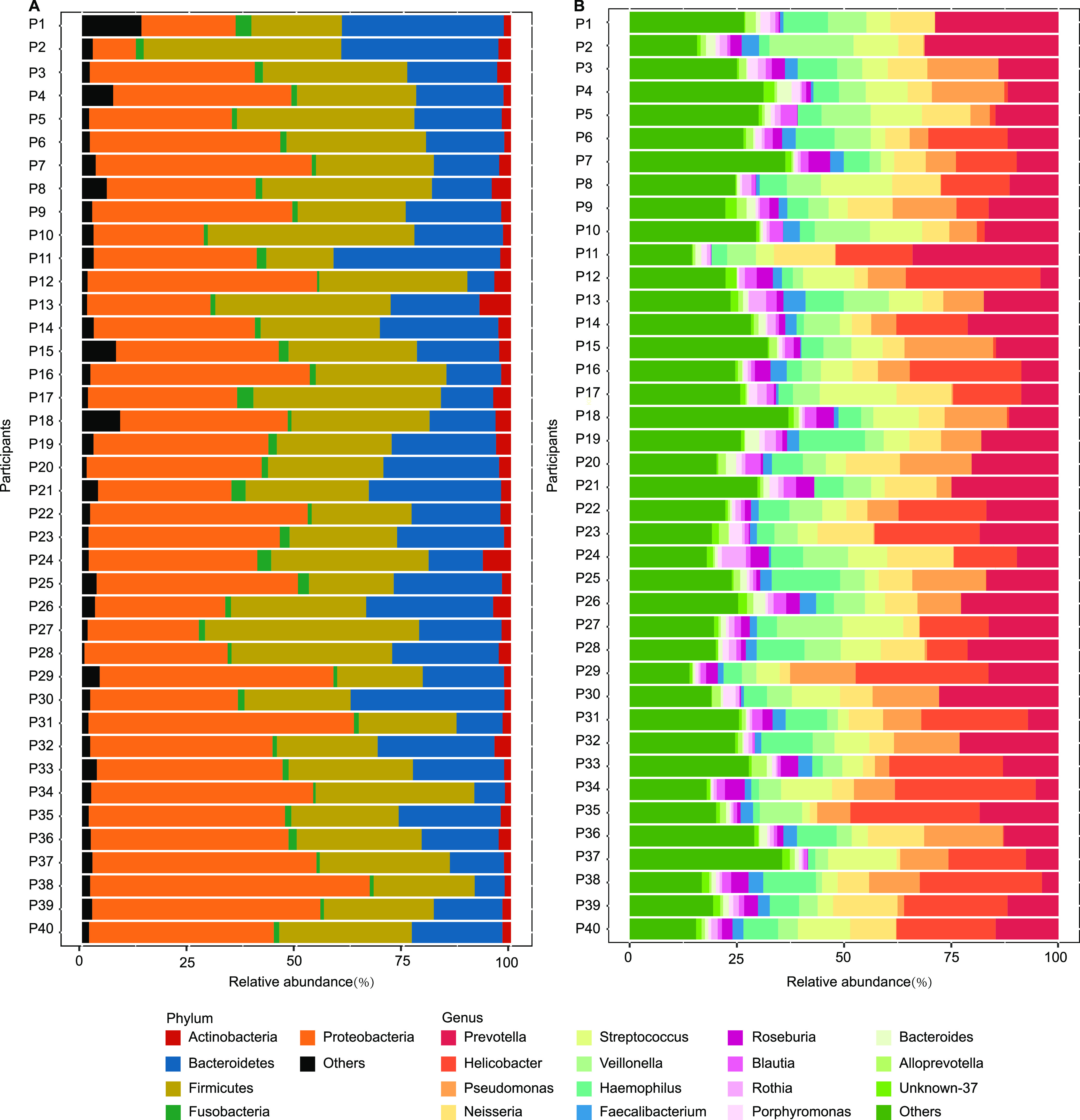
Interindividual average relative abundances at phylum and genus levels. (A) Interindividual average relative abundances (%) at the phylum level. For each phylum in each participant, the average relative abundance (%) was the average of this phylum in six sites. (B) Interindividual average relative abundances (%) at the genus level. For each genus in each participant, the average relative abundance (%) was the average of this genus in six GI sites.

Between participants, there was microbial similarity both in observed ASVs (*P *> 0.05) and Shannon index values (*P *> 0.05). CPCoA analysis shows that the six specimens from each participant had large overlaps (*P *> 0.05), but only 14% of the variance could be explained. Based on Bray-Curtis distances, there was no interparticipant or intraparticipant difference (*P *< 0.05). The microbial compositions of the dominant phyla, including *Actinobacteria*, *Bacteroidetes*, *Firmicutes*, *Fusobacteria*, and *Proteobacteria*, were similar between participants (Fig. 5A). At the genus level, the average relative abundances of the top 15 genera from the highest to the lowest were as follows: Prevotella (15.87%, *P *= 0.16), Helicobacter (12.36%, *P *= 0.56), Pseudomonas (8.06%, *P *= 0.97), Neisseria (7.89%, *P *= 0.81), Streptococcus (7.57%, *P *= 0.90), Veillonella (6.65%, *P *= 0.57), Haemophilus (6.42%, *P *= 0.87), Faecalibacterium (2.07%, *P *= 0.98), Roseburia (1.97%, *P *= 1.00), Blautia (1.57%, *P *= 1.00), Rothia (1.24%, *P *= 0.93), Porphyromonas (1.08%, *P *= 0.68), Bacteroides (1.07%, *P *= 0.18), and Alloprevotella (1.02%, *P *= 0.60) (Fig. 5B). Generally, the microbial characteristics between participants were consistent with the microbial characteristics within participants, which were diverse and dynamic.

## DISCUSSION

The microbial characteristics in each GI site are closely related to the anatomical structure, and the microbial compositions overlap in the descending GI tract (15, 21, 22). In this study, there were great differences in the microbial compositions of the upper and lower GI tract. Each GI site had its own microbial characteristics that overlapped those of adjacent sites, and differences and commonalities coexisted in the microbial compositions of different sites. This study provides an opportunity to understand the microbial diversity and similarity in different GI sites in Chinese individuals.

The oral cavity, as the initial gateway of the GI tract, is constantly exposed to both inhaled and ingested microbes and has more than 700 species or phylotypes in its microbiota (23), which explains the highest observed ASVs in saliva in this study. With almost 10^11^ bacterial cells moving from the mouth into the stomach per day (15, 24), the observed ASVs decreased accordingly. They dropped to their lowest point in gastric juice, which was contrary to the findings of Sung et al. (16). Sung et al. (16) et al. found that the mean number of operational taxonomic units (OTUs) was higher in gastric fluid than in gastric mucosa. The disease status of enrolled participants and the sampling sites in the noncardia stomach in the two studies are probable influencing factors. Surprisingly, this study found that the alpha diversity in feces was lower than expected. A previous study showed that the diversity was lower in feces than in saliva (25). The distinct discrepancy in microbial communities between the UGI tract and feces and the dominant role of the characteristic genera in the fecal microbiota may be a reasonable explanation for the lower diversity in feces than saliva. Microbial DNA in different GI sites was extracted by the same protocol in this study, which may be another explanation and needs to be further explored in the future. Additionally, the acid environment influenced the microbial diversity in gastric juice very strongly, which may also explain the surprising upward tendency of alpha diversity from gastric juice to feces in this study (15, 25–27).

In this study, compared to the microbial communities of the cardia biopsy, noncardia biopsy, gastric juice, and fecal specimens, the microbial community in saliva specimens was comparatively similar to that of esophageal swab specimens, which is consistent with the findings of Fillon et al. (28) but inconsistent with those of Dong et al. (13). To answer the inconsistencies between this study and Dong’s, the various sampling sites in this study were important explanations for the relevant microbial similarities between saliva and esophageal swab specimens. Neisseria, Prevotella, Streptococcus, and Haemophilus have been characterized in both the oral cavity and the esophagus (13, 29–31). This study found that Neisseria and Prevotella were saliva-specific genera, which was similar to the findings of Segata et al. (15). However, a study (13) comparing three sites in the oral cavity only indicated Neisseria as a saliva-specific genus, and the relative abundances of Neisseria in saliva and tongue dorsum specimens were higher than its abundance in supragingival plaque specimens. Streptococcus and Haemophilus were esophagus-specific genera in this study. The average relative abundances of Streptococcus decreased from esophagus to cardia to noncardia specimens, which is consistent with the findings of Wurm et al. (32). Previous studies have indicated that decreased levels of Streptococcus in the esophagus are associated with gastroesophageal reflux disease (GERD) (33), Barrett’s esophagus (BE) (22), esophageal adenocarcinoma (EAC) (22), and esophageal squamous cell carcinoma (ESCC) (34, 35) and an enrichment of Haemophilus is relevant to eosinophilic esophagitis (EoE) (33). More significant, the combination of Streptococcus and Prevotella (34, 36) or Neisseria (37) is emerging in the exploration of the relationship between microbiota and esophageal health or disease.

The gastric microbiota, one of the important components of the gut microbiota, can be shaped by the host physiology as well as by external factors, such as H. pylori infection, proton pump inhibitors (PPIs), and antibiotics (38). In this study, the microbial diversity was lower in the H. pylori-positive group than in the H. pylori-negative group in both cardia and noncardia biopsy specimens. However, there have been different conclusions. Bik et al., Khosravi et al., and Maldonado-Contreras et al. found that the presence of H. pylori did not significantly modify the diversity of gastric microbiota, while Andersson et al. found higher diversity in participants without H. pylori infection than in participants with H. pylori infection (19, 39–41). All participants in the Andersson et al. study were healthy, while all or some participants in the other studies had GI disease. In detail, there was a vicious circle from H. pylori infection to disease. With the progression of disease, the initial influence of H. pylori on other, non-H. pylori growth gradually weakened, and the microbial composition gradually became balanced. One supposition is that the inclusion of cardia biopsy specimens may play an important role in the explanation. As we all know, H. pylori normally inhabits the mucus-lined surface of the gastric corpus and antrum. H. pylori infection is identified as the strongest risk factor for gastric cancer and has a higher risk ratio for noncardia cancer than for cardia cancer (42). Consequently, it is reasonable that the impact of H. pylori on the noncardia microbiota is more significant than its impact on the cardia microbiota. Besides, the results of this study indicated that the microbial diversity was always higher in cardia than in noncardia biopsy specimens, no matter the H. pylori infection status; the acid environment in the stomach may be a reasonable explanation, but there are limited relevant studies. Additionally, the relative abundance of Helicobacter in cardia specimens was second only to its abundance in noncardia specimens, and Streptococcus and Haemophilus were richer in cardia than in noncardia specimens but lower than in esophagus specimens, which reflected the boundary (esophagogastric junction) microbial characteristics of cardia.

After H. pylori colonizes the stomach mucosa, it becomes the predominant species of the gastric microbiota. H. pylori is a spiral-shaped, microaerophilic, Gram-negative bacterium with flagella that has urease, catalase, and oxidase activity (43). Every H. pylori carrier will ultimately develop chronic gastritis. In turn, the inflammatory response damages the gastric epithelial lining and allows H. pylori to flourish. Then, H. pylori infection worsens, gastritis results, and acid-secreting glands decrease, leading to an increase in the pH of gastric juice (44). However, the diversities in the HP group, the H group, and the P group were similar to each other, and the HP group was relevantly higher. This suggests that H. pylori infection might not affect stomach acid secretion as much, since the participants in this study did not have gastritis. The relative abundances of Pseudomonas and Acinetobacter increased from abnormal (in the HP group) to normal (in the LN group), while the change in the relative abundance of Rothia was the converse, which was similar to the change of alpha diversity in gastric juice specimens. Besides, Pseudomonas was the characteristic genus of gastric juice in this study. There is a study from Venezuela reporting that Pseudomonas strains may interfere with the identification of H. pylori (45). However, less attention has been paid to Pseudomonas until now, especially in gastric juice.

Apart from anatomical sites, individual differences play an important role in the microbial composition. Gall et al. found that microbial communities in UGI sites displayed higher interindividual variation but overlapping community membership between sites (22), and in the upper and lower gut, the intra- and interindividual mucosa-associated microbiota profiles were significantly different in healthy Japanese (27). Hisada et al. found that interindividual variations were significantly larger than intraindividual variations for most of the dominant genera in fecal samples (46). Individual difference is an important and nonnegligible factor for microbial studies. In this study, inter- and intraparticipant microbial similarities were shown based on the Bray-Curtis distances. The variances in study design among the above-mentioned studies are potential reasons for the opposite conclusions described, especially the collection sites, sample types, and sampling methods. Specifically, all samples in the studies of Gall et al. (22) and Kashiwagi et al. (27) were collected via brush sampling, while this study included saliva, tissue biopsy, mucosal swab, gastric juice, and fecal specimens. Additionally, gastrointestinal microbiota can be influenced by various factors, including geographic origin, host genotype, and diet (47). Two microbial studies (48, 49) based in several provinces in China found that the microbial composition in feces was mainly influenced by geography. Previous studies (50–52) based in one or two provinces in China have shown similar patterns in the bacterial, fungal, and viral components. In this study, all participants were from the same village in Linzhou County and had no statistical differences in diet and lifestyle, which effectively reduced the heterogeneity.

This study plays a fundamental role in mapping the microbial composition in Chinese participants, which provides the scientific basis for exploring the gastrointestinal-host microbiota relationship in the future. However, there were several limitations in this study. First, due to the strict inclusion criteria and complicated collection procedures, this exploratory study was based on the UGI screening project in China, which ensured the feasibility and reliability of study implementation, while the sample size was small. Second, H. pylori infection was one of the main factors discussed in this study. However, only one biopsy specimen was collected from the noncardia stomach, which made it hard to testify as to the initial infection site and colonization status of H. pylori. Third, we used a fecal occult blood test to determine lower GI disease status in this study, but there was no solid pathological diagnosis, and the fecal occult blood tests of all enrolled participants in this study were negative. Finally, due to the similarity of epidemiological factors and the small sample size, this study did not analyze the interaction between microbiota and epidemiological factors.

### Conclusion.

Simply put, differences and commonalities coexist in the microbial composition of different GI sites. Within the individual, each phylum or genus may exist in more than one site, while each site has its own relatively characteristic microbiota. This study provides baseline microbial information, especially for the UGI microbiota, which will help to deepen our understanding of microbial characteristics in the GI tract and then gradually cover the fields of etiology, primary screening, and treatment. However, the relationship between microbiota and epidemiological factors is still unclear. For this, multidisciplinary studies with multicenter cooperation, larger sample sizes, multiple types of specimens, and prospective studies need to be carried out in the future.

## MATERIALS AND METHODS

### Study participants.

**FIG 6:**
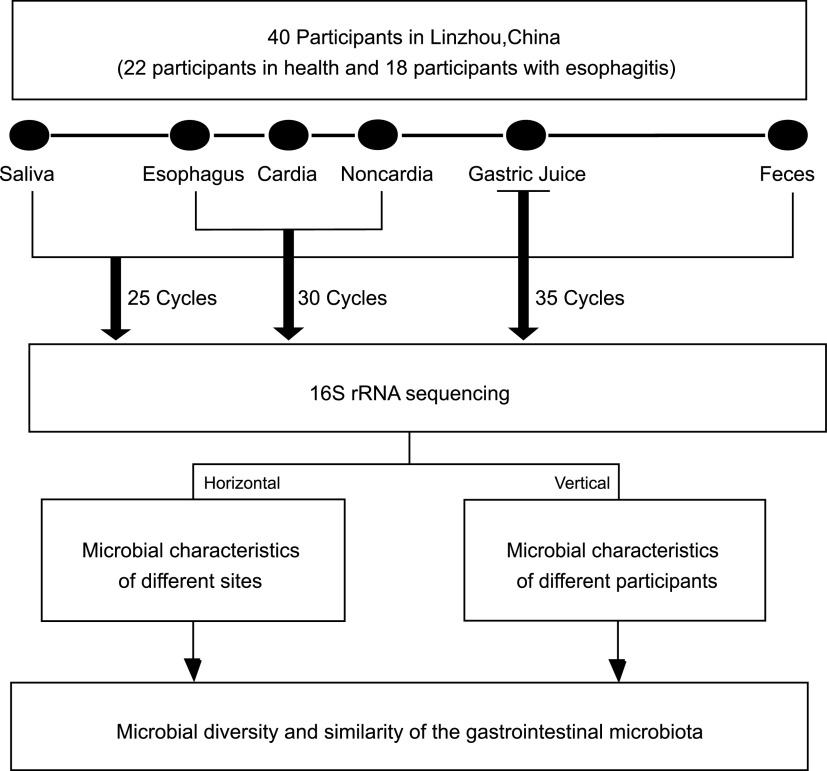
Flowchart of participant enrollment and sample collection.

We recruited 40 participants aged 40 to 69 years old at the Linzhou Cancer Hospital in Henan Province in China (Fig. 6). The healthy group included 22 participants that did not have esophageal, cardia, or noncardia stomach lesions, and the esophagitis group included 18 participants that had mild or moderate esophagitis and no lesions in the cardia and noncardia stomach. All participants were local residents who were screened for UGI cancer in August in 2019. All participants had no major metabolic or systemic disease and had negative results in fecal occult blood testing. None of the participants had taken antibiotics or proton pump inhibitors in the past month. Epidemiologic information was collected by trained staff. All participants were clearly informed and signed written informed consent. This study was approved and overseen by the Institutional Review Board of the Cancer Hospital of Chinese Academy of Medical Sciences.

### Sample collection.

Saliva, esophageal swab, cardia biopsy, noncardia biopsy, gastric juice, and fecal specimens were collected from each participant during the UGI cancer screening. Fresh feces were collected before endoscopic examination by disposable plastic box within 5 min after emission and then smeared on FOBT cards (fecal occult blood test cards; Baso-Diagnostics, Inc., Zhuhai, China) by the same trained staff. After mouth rinsing, saliva was collected in the salivary DNA collection device (saliva DNA sample collection kit; Zeesan Biotech Co., Xiamen, China). Other specimens were collected during the endoscopy examination (no defoaming agent was used before endoscopy examination). First, 15 mL gastric juice was absorbed into the collection device (Polyp Trap; Micro-Tech Co., Ltd., Nanjing, China). Next, cardia and noncardia biopsy specimens were dissected by an experienced endoscopy physician with sterile forceps under strict procedures and then placed into sterile tubes (2.0-mL cryogenic vials; Corning Incorporated, New York, NY, USA). Esophageal swab specimens were collected using sterile swabs (disposable cell brush; Jiuhong Medical Instrument Co., Changzhou, China). If there were lesions, 5 looping brushes were taken at the lesion, and if not, 5 looping brushes were taken at the middle esophagus. After swab collection, the swab head was cut off into a sterile tube (3.0-mL cryogenic tube; Simport Scientific, Beloeil, Canada) containing 1.5 mL of cell-preserving fluid (ThinPrep PreservCyt solution; Hologic, San Diego, CA, USA). All specimens were stored at −80°C immediately after sampling. After all specimens were obtained, we transferred the specimens to the laboratory with dry ice by express delivery. Before DNA extraction, no other processes were performed on specimens.

### Gastric pH test, H. pylori test, and fecal occult blood test.

After the absorption of gastric juice, pH test strips (pH-fix 0.0–6.0; Macherey-Nagel GmbH & Co. KG, Düren, Germany) were used for pH testing. All pH tests were performed by the same staff to reduce bias in the color comparisons. The noncardia biopsy specimen from each participant was subjected to Helicobacter pylori testing by rapid urease test based on the UGI screening project. The fresh biopsy specimen was placed into the substrate reaction solution with a bamboo stick, which was fully oscillated and placed for around 5 min at room temperature. H. pylori testing and result determination were conducted by the same staff. Additionally, fecal occult blood testing was performed by the pyramidon semiquantitative method (fecal occult blood reagent; Baso Diagnostics, Inc., Zhuhai, China) or a home self-testing device (Hangzhou New Horizon Health Technology Co., Ltd., Hangzhou, China).

### Bacterial DNA extraction, amplification, and sequencing.

Bacterial DNA in all specimens was extracted based on the protocol in the study of Manichanh et al. (53) and stored in Tris-EDTA buffer solution at −80°C prior to other processes. DNA amplicons covering the V4 region were generated using the following primers: 515F (5′-GTGYCAGCMGCCGCGGTAA-3′) and 806R (5′-GGACTACNVGGGTWTCTAAT-3′) (54, 55). The PCR mixtures contained 1 μL of forward and reverse primer (10 μmol/L), 1 μL of template DNA, 4 μL of deoxyribonucleoside triphosphates (dNTPs) (2.5 mmol/L), 5 μL of 10× EasyPfu buffer, 1 μL of EasyPfu DNA polymerase (2.5 U/μL), and 1 μL of double-distilled water in a 50-μL reaction mixture volume. The PCR thermal cycling consisted of an initial denaturation step at 95°C for 5 min, followed by cycles of denaturation at 94°C for 30 s, annealing at 60°C for 30 s, and extension at 72°C for 40 s, with a final extension step at 72°C for 4 min. The numbers of cycles were as follows: 25 cycles for saliva and fecal specimens, 30 cycles for swab and biopsy specimens, and 35 cycles for gastric juice specimens (Fig. 6) Amplicons from each specimen were run on an agarose gel to ensure consistent sequencing length. Amplicons were quantified with the Qubit double-stranded DNA (dsDNA) high-sensitivity (HS) assay kit (catalog no. Q32854; Thermo Fisher Scientific/Invitrogen, Waltham, MA, USA). Then, the amplicon libraries were pooled at an equal mass of 100 ng per sample and were sequenced on the Illumina MiniSeq platform. In the DNA extraction, PCR amplification, and sequencing steps, positive and negative quality controls were included along with the study samples in each batch. The positive quality control was a fecal sample that had been deeply sequenced previously, and the negative control was a sample of water without DNA or contamination.

### Sequence processing and taxonomic classification.

In this study, 240 specimens were successfully sequenced. Fecal samples were sequenced with more than 30,000 reads, while other samples depended on the actual situation. The common quality control process included removal of low-quality, artifactual, and chimeric reads, sequencing error corrections, and read-length trimming. The DADA2 algorithm was used to remove possible phiX reads and chimeric sequences, correct sequencing errors, and generate amplicon sequence variants (ASVs) (56). To avoid sequencing errors at the ends, the remaining reads were truncated from 0 to 150 bp. Paired-end reads were connected at the minimum overlap length of 20 bp between the forward and reverse reads, and maximum 2-bp mismatches were allowed inside the overlap zone. Sequence data were processed using the Quantitative Insights into Microbial Ecology (QIIME2) platform (57). Taxonomic assignment of the ASVs was determined based on a pretrained naive Bayes classifier (trained on the SILVA database [58, 59]) via the q2-feature-classifier plugin. To avoid sampling depth bias, 4,000 reads were randomly selected from each sample when calculating the relative abundances of ASVs and taxa.

### Group definition.

Several grouping methods were used in this study, as follows. (i) For GI site, based on the specimen collection site, specimens were assigned to the saliva group, esophagus group, cardia group, noncardia group, gastric juice group, or feces group. (ii) For H. pylori infection, participants were divided into the positive (P) group or the negative (N) group. (iii) For gastric pH, depending on whether the pH value of gastric juice was lower than or higher than 2, participants were divided into the lower (L) group or the higher (H) group. (iv) For H. pylori infection and gastric pH, the groups included the HP group (higher pH and H. pylori positive), the LP group (lower pH and H. pylori positive), the HN group (higher pH and H. pylori negative), and the LN group (lower pH and H. pylori negative).

### Statistical analysis.

Epidemiological factors (Table 1) were compared between healthy participants and participants with esophagitis by the chi-square test or Fisher’s test. Alpha diversities (observed ASVs and Shannon index) were compared between two groups by the Wilcoxon test and among more than two groups by the Kruskal-Wallis test. After the Kruskal-Wallis test, Dunn’s test was used for multiple testing (Pairwise Multiple Comparisons of Mean Rank Sums [PMCMR] package). Beta diversity analysis was based on Bray-Curtis distance or weighted UniFrac distance. If there were more than three groups, constrained principal coordinate analysis (CPCoA) was performed for beta diversity, or else principal coordinate analysis (PCoA) was performed (Analyses of Phylogenetics and Evolution [APE] package). The adonis test was performed after PCoA (Vegan package). Microbial compositions at the phylum and genus levels were described and compared using the Kruskal-Wallis test or the Wilcoxon test. Additionally, SourceTracker was performed as described in Knights et al., where the fecal specimens were sink specimens (60). Then, distance-based redundancy analysis (db-RDA) and linear discriminant analysis effect size (LEfSe) were conducted by using the Vegan package and Galaxy website to explore the characteristic genus. In addition, correlation analysis was performed for the dominant genera (Corrplot package). Moreover, functional profiling of the GI microbiota was performed using the PICRUSt2 algorithm (61). For each sample, the composition of KEGG orthologs (KOs) was predicted based on the functional information on the reference ASVs (62). KEGG pathway compositions were generated according to the assignment of KOs. Between interindividual and intraindividual data, the Wilcoxon test was used to compare the microbial diversities based on Bray-Curtis distance. A conventional level of significance (*P *< 0.05) was used for rejecting the null hypothesis. The Bonferroni correction was used to adjust *P* values in multiple testing. All statistical analysis was performed with R Studio (version 1.1.456).

### Ethics declaration.

This study was approved and overseen by the Institutional Review Board of the Cancer Hospital of Chinese Academy of Medical Sciences.

### Data availability.

The sequencing data are available in the Genome Sequence Archive (GSA) database under accession number CRA004966.

## References

[1] Requena T, Martínez-Cuesta MC, Peláez P. 2018. Diet and microbiota linked in health and disease. Food Funct 9:688–704. doi:10.1039/c7fo01820g. 29410981

[2] Belkaid Y, Harrison OJ. 2017. Homeostatic immunity and the microbiota. Immunity 46:562–576. doi:10.1016/j.immuni.2017.04.008. 28423337 PMC5604871

[3] Dominguez-Bello MG, Godoy-Vitorino F, Knight R, Blaser MJ. 2019. Role of the microbiome in human development. Gut 68:1108–1114. doi:10.1136/gutjnl-2018-317503. 30670574 PMC6580755

[4] Garrett WS. 2015. Cancer and the microbiota. Science 348:80–86. doi:10.1126/science.aaa4972. 25838377 PMC5535753

[5] Tilg H, Moschen AR. 2014. Microbiota and diabetes: an evolving relationship. Gut 63:1513–1521. doi:10.1136/gutjnl-2014-306928. 24833634

[6] Maruvada P, Leone V, Kaplan LM, Chang EB. 2017. The human microbiome and obesity: moving beyond associations. Cell Host Microbe 22:589–599. doi:10.1016/j.chom.2017.10.005. 29120742

[7] Vogtmann E, Goedert JJ. 2016. Epidemiologic studies of the human microbiome and cancer. Br J Cancer 114:237–242. doi:10.1038/bjc.2015.465. 26730578 PMC4742587

[8] Foster JA, McVey Neufeld K-A. 2013. Gut-brain axis: how the microbiome influences anxiety and depression. Trends Neurosci 36:305–312. doi:10.1016/j.tins.2013.01.005. 23384445

[9] Yamamura K, Izumi D, Kandimalla R, Sonohara F, Baba Y, Yoshida N, Kodera Y, Baba H, Goel A. 2019. Intratumoral Fusobacterium nucleatum levels predict therapeutic response to neoadjuvant chemotherapy in esophageal squamous cell carcinoma. Clin Cancer Res 25:6170–6179. doi:10.1158/1078-0432.CCR-19-0318. 31358543 PMC6801075

[10] Yamaoka Y, Suehiro Y, Hashimoto S, Hoshida T, Fujimoto M, Watanabe M, Imanaga D, Sakai K, Matsumoto T, Nishioka M, Takami T, Suzuki N, Hazama S, Nagano H, Sakaida I, Yamasaki T. 2018. Fusobacterium nucleatum as a prognostic marker of colorectal cancer in a Japanese population. J Gastroenterol 53:517–524. doi:10.1007/s00535-017-1382-6. 28823057

[11] Vindigni SM, Surawicz CM. 2017. Fecal microbiota transplantation. Gastroenterol Clin North Am 46:171–185. doi:10.1016/j.gtc.2016.09.012. 28164849

[12] Tong X, Xu J, Lian F, Yu X, Zhao Y, Xu L, Zhang M, Zhao X, Shen J, Wu S, Pang X, Tian J, Zhang C, Zhou Q, Wang L, Pang B, Chen F, Peng Z, Wang J, Zhen Z, Fang C, Li M, Chen L, Zhao L. 2018. Structural alteration of gut microbiota during the amelioration of human type 2 diabetes with hyperlipidemia by metformin and a traditional Chinese herbal formula: a multicenter, randomized, open label clinical trial. mBio 9:e02392-17. doi:10.1128/mBio.02392-17. 29789365 PMC5964358

[13] Dong L, Yin J, Zhao J, Ma S-R, Wang H-R, Wang M, Chen W, Wei W-Q. 2018. Microbial similarity and preference for specific sites in healthy oral cavity and esophagus. Front Microbiol 9:1603. doi:10.3389/fmicb.2018.01603. 30065718 PMC6056649

[14] Liu A-Q, Vogtmann E, Shao D-T, Abnet CC, Dou H-Y, Qin Y, Su Z, Wei W-Q, Chen W. 2019. A comparison of biopsy and mucosal swab specimens for examining the microbiota of upper gastrointestinal carcinoma. Cancer Epidemiol Biomarkers Prev 28:2030–2037. doi:10.1158/1055-9965.EPI-18-1210. 31519703 PMC7294753

[15] Segata N, Haake SK, Mannon P, Lemon KP, Waldron L, Gevers D, Huttenhower C, Izard J. 2012. Composition of the adult digestive tract bacterial microbiome based on seven mouth surfaces, tonsils, throat and stool samples. Genome Biol 13:R42. doi:10.1186/gb-2012-13-6-r42. 22698087 PMC3446314

[16] Sung J, Kim N, Kim J, Jo HJ, Park JH, Nam RH, Seok Y-J, Kim Y-R, Lee DH, Jung HC. 2016. Comparison of gastric microbiota between gastric juice and mucosa by next generation sequencing method. J Cancer Prev 21:60–65. doi:10.15430/JCP.2016.21.1.60. 27051651 PMC4819668

[17] Arnold M, Abnet CC, Neale RE, Vignat J, Giovannucci EL, McGlynn KA, Bray F. 2020. Global burden of 5 major types of gastrointestinal cancer. Gastroenterology 159:335–349. doi:10.1053/j.gastro.2020.02.068. 32247694 PMC8630546

[18] Chen R, Liu Y, Song G, Li B, Zhao D, Hua Z, Wang X, Li J, Hao C, Zhang L, Liu S, Wang J, Zhou J, Zhang Y, Li B, Li Y, Feng X, Li L, Dong Z, Wei W, Wang G. 2021. Effectiveness of one-time endoscopic screening programme in prevention of upper gastrointestinal cancer in China: a multicentre population-based cohort study. Gut 70:251–260. doi:10.1136/gutjnl-2019-320200. 32241902 PMC7815635

[19] Bik EM, Eckburg PB, Gill SR, Nelson KE, Purdom EA, Francois F, Perez-Perez G, Blaser MJ, Relman DA. 2006. Molecular analysis of the bacterial microbiota in the human stomach. Proc Natl Acad Sci USA 103:732–737. doi:10.1073/pnas.0506655103. 16407106 PMC1334644

[20] Schulz C, Schütte K, Koch N, Vilchez-Vargas R, Wos-Oxley ML, Oxley APA, Vital M, Malfertheiner P, Pieper DH. 2018. The active bacterial assemblages of the upper GI tract in individuals with and without Helicobacter infection. Gut 67:216–225. doi:10.1136/gutjnl-2016-312904. 27920199

[21] Norder Grusell E, Dahlén G, Ruth M, Ny L, Quiding-Järbrink M, Bergquist H, Bove M. 2013. Bacterial flora of the human oral cavity, and the upper and lower esophagus. Dis Esophagus 26:84–90. doi:10.1111/j.1442-2050.2012.01328.x. 22394217

[22] Gall A, Fero J, McCoy C, Claywell BC, Sanchez CA, Blount PL, Li X, Vaughan TL, Matsen FA, Reid BJ, Salama NR. 2015. Bacterial composition of the human upper gastrointestinal tract microbiome is dynamic and associated with genomic instability in a Barrett’s esophagus cohort. PLoS One 10:e0129055. doi:10.1371/journal.pone.0129055. 26076489 PMC4468150

[23] Aas JA, Paster BJ, Stokes LN, Olsen I, Dewhirst FE. 2005. Defining the normal bacterial flora of the oral cavity. J Clin Microbiol 43:5721–5732. doi:10.1128/JCM.43.11.5721-5732.2005. 16272510 PMC1287824

[24] Socransky SS, Haffajee AD. 2002. Dental biofilms: difficult therapeutic targets. Periodontol 2000 28:12–55. doi:10.1034/j.1600-0757.2002.280102.x. 12013340

[25] Costello EK, Lauber CL, Hamady M, Fierer N, Gordon JI, Knight R. 2009. Bacterial community variation in human body habitats across space and time. Science 326:1694–1697. doi:10.1126/science.1177486. 19892944 PMC3602444

[26] Vasapolli R, Schütte K, Schulz C, Vital M, Schomburg D, Pieper DH, Vilchez-Vargas R, Malfertheiner P. 2019. Analysis of transcriptionally active bacteria throughout the gastrointestinal tract of healthy individuals. Gastroenterology 157:1081–1092.e3. doi:10.1053/j.gastro.2019.05.068. 31175864

[27] Kashiwagi S, Naito Y, Inoue R, Takagi T, Nakano T, Inada Y, Fukui A, Katada K, Mizushima K, Kamada K, Uchiyama K, Handa O, Ishikawa T, Itoh Y. 2020. Mucosa-associated microbiota in the gastrointestinal tract of healthy Japanese. Digestion 101:107–120. doi:10.1159/000496102. 30721900

[28] Fillon SA, Harris JK, Wagner BD, Kelly CJ, Stevens MJ, Moore W, Fang R, Schroeder S, Masterson JC, Robertson CE, Pace NR, Ackerman SJ, Furuta GT. 2012. Novel device to sample the esophageal microbiome—the esophageal string test. PLoS One 7:e42938. doi:10.1371/journal.pone.0042938. 22957025 PMC3434161

[29] Pei Z, Bini EJ, Yang L, Zhou M, Francois F, Blaser MJ. 2004. Bacterial biota in the human distal esophagus. Proc Natl Acad Sci USA 101:4250–4255. doi:10.1073/pnas.0306398101. 15016918 PMC384727

[30] Yang L, Lu X, Nossa CW, Francois F, Peek RM, Pei Z. 2009. Inflammation and intestinal metaplasia of the distal esophagus are associated with alterations in the microbiome. Gastroenterology 137:588–597. doi:10.1053/j.gastro.2009.04.046. 19394334 PMC2963147

[31] Park CH, Lee SK. 2020. Exploring esophageal microbiomes in esophageal diseases: a systematic review. J Neurogastroenterol Motil 26:171–179. doi:10.5056/jnm19240. 32235026 PMC7176507

[32] Wurm P, Dörner E, Kremer C, Spranger J, Maddox C, Halwachs B, Harrison U, Blanchard T, Haas R, Högenauer C, Gorkiewicz G, Fricke WF. 2018. Qualitative and quantitative DNA- and RNA-based analysis of the bacterial stomach microbiota in humans, mice, and gerbils. mSystems 3:e00262-18. doi:10.1128/mSystems.00262-18. 30505943 PMC6247015

[33] Harris JK, Fang R, Wagner BD, Choe HN, Kelly CJ, Schroeder S, Moore W, Stevens MJ, Yeckes A, Amsden K, Kagalwalla AF, Zalewski A, Hirano I, Gonsalves N, Henry LN, Masterson JC, Robertson CE, Leung DY, Pace NR, Ackerman SJ, Furuta GT, Fillon SA. 2015. Esophageal microbiome in eosinophilic esophagitis. PLoS One 10:e0128346. doi:10.1371/journal.pone.0128346. 26020633 PMC4447451

[34] Liu Y, Lin Z, Lin Y, Chen Y, Peng X-E, He F, Liu S, Yan S, Huang L, Lu W, Xiang Z, Hu Z. 2018. Streptococcus and Prevotella are associated with the prognosis of oesophageal squamous cell carcinoma. J Med Microbiol 67:1058–1068. doi:10.1099/jmm.0.000754. 29923815

[35] Shao D, Vogtmann E, Liu A, Qin J, Chen W, Abnet CC, Wei W. 2019. Microbial characterization of esophageal squamous cell carcinoma and gastric cardia adenocarcinoma from a high-risk region of China. Cancer 125:3993–4002. doi:10.1002/cncr.32403. 31355925 PMC7285383

[36] Deshpande NP, Riordan SM, Castano-Rodriguez N, Wilkins MR, Kaakoush NO. 2018. Signatures within the esophageal microbiome are associated with host genetics, age, and disease. Microbiome 6:227. doi:10.1186/s40168-018-0611-4. 30558669 PMC6297961

[37] Li M, Shao D, Zhou J, Gu J, Qin J, Chen W, Wei W. 2020. Signatures within esophageal microbiota with progression of esophageal squamous cell carcinoma. Chin J Cancer Res 32:755–767. doi:10.21147/j.issn.1000-9604.2020.06.09. 33446998 PMC7797230

[38] Wu WM, Yang YS, Peng LH. 2014. Microbiota in the stomach: new insights. J Dig Dis 15:54–61. doi:10.1111/1751-2980.12116. 24245792

[39] Khosravi Y, Dieye Y, Poh BH, Ng CG, Loke MF, Goh KL, Vadivelu J. 2014. Culturable bacterial microbiota of the stomach of Helicobacter pylori positive and negative gastric disease patients. Sci World J 2014:610421. doi:10.1155/2014/610421. PMC4106172 25105162

[40] Maldonado-Contreras A, Goldfarb KC, Godoy-Vitorino F, Karaoz U, Contreras M, Blaser MJ, Brodie EL, Dominguez-Bello MG. 2011. Structure of the human gastric bacterial community in relation to Helicobacter pylori status. ISME J 5:574–579. doi:10.1038/ismej.2010.149. 20927139 PMC3105737

[41] Andersson AF, Lindberg M, Jakobsson H, Backhed F, Nyren P, Engstrand L. 2008. Comparative analysis of human gut microbiota by barcoded pyrosequencing. PLoS One 3:e2836. doi:10.1371/journal.pone.0002836. 18665274 PMC2475661

[42] Cavaleiro-Pinto M, Peleteiro B, Lunet N, Barros H. 2011. Helicobacter pylori infection and gastric cardia cancer: systematic review and meta-analysis. Cancer Causes Control 22:375–387. doi:10.1007/s10552-010-9707-2. 21184266

[43] Camilo V, Sugiyama T, Touati E. 2017. Pathogenesis of Helicobacter pylori infection. Helicobacter 22:e12405. doi:10.1111/hel.12405. 28891130

[44] Park YH, Kim N. 2015. Review of atrophic gastritis and intestinal metaplasia as a premalignant lesion of gastric cancer. J Cancer Prev 20:25–40. doi:10.15430/JCP.2015.20.1.25. 25853101 PMC4384712

[45] Domínguez-Bello MG, Reyes N, Teppa-Garrán A, Romero R. 2000. Interference of Pseudomonas strains in the identification of Helicobacter pylori. J Clin Microbiol 38:937. doi:10.1128/JCM.38.2.937-937.2000. 10722321 PMC86256

[46] Hisada T, Endoh K, Kuriki K. 2015. Inter- and intra-individual variations in seasonal and daily stabilities of the human gut microbiota in Japanese. Arch Microbiol 197:919–934. doi:10.1007/s00203-015-1125-0. 26068535 PMC4536265

[47] Bäckhed F, Fraser CM, Ringel Y, Sanders ME, Sartor RB, Sherman PM, Versalovic J, Young V, Finlay BB. 2012. Defining a healthy human gut microbiome: current concepts, future directions, and clinical applications. Cell Host Microbe 12:611–622. doi:10.1016/j.chom.2012.10.012. 23159051

[48] Zhang J, Guo Z, Xue Z, Sun Z, Zhang M, Wang L, Wang G, Wang F, Xu J, Cao H, Xu H, Lv Q, Zhong Z, Chen Y, Qimuge S, Menghe B, Zheng Y, Zhao L, Chen W, Zhang H. 2015. A phylo-functional core of gut microbiota in healthy young Chinese cohorts across lifestyles, geography and ethnicities. ISME J 9:1979–1990. doi:10.1038/ismej.2015.11. 25647347 PMC4542028

[49] Lu J, Zhang L, Zhai Q, Zhao J, Zhang H, Lee Y-K, Lu W, Li M, Chen W. 2021. Chinese gut microbiota and its associations with staple food type, ethnicity, and urbanization. NPJ Biofilms Microbiomes 7:71. doi:10.1038/s41522-021-00245-0. 34489454 PMC8421333

[50] Winglee K, Howard AG, Sha W, Gharaibeh RZ, Liu J, Jin D, Fodor AA, Gordon-Larsen P. 2017. Recent urbanization in China is correlated with a westernized microbiome encoding increased virulence and antibiotic resistance genes. Microbiome 5:121. doi:10.1186/s40168-017-0338-7. 28915922 PMC5603068

[51] Sun Y, Zuo T, Cheung CP, Gu W, Wan Y, Zhang F, Chen N, Zhan H, Yeoh YK, Niu J, Du Y, Zhang F, Wen Y, Yu J, Sung JJY, Chan PKS, Chan FKL, Wang K, Ng SC, Miao Y. 2021. Population-level configurations of gut mycobiome across 6 ethnicities in urban and rural China. Gastroenterology 160:272–286. doi:10.1053/j.gastro.2020.09.014. 32956679

[52] Zuo T, Sun Y, Wan Y, Yeoh YK, Zhang F, Cheung CP, Chen N, Luo J, Wang W, Sung JJY, Chan PKS, Wang K, Chan FKL, Miao Y, Ng SC. 2020. Human-gut-DNA virome variations across geography, ethnicity, and urbanization. Cell Host Microbe 28:741–751. doi:10.1016/j.chom.2020.08.005. 32910902

[53] Manichanh C, Rigottier-Gois L, Bonnaud E, Gloux K, Pelletier E, Frangeul L, Nalin R, Jarrin C, Chardon P, Marteau P, Roca J, Dore J. 2006. Reduced diversity of faecal microbiota in Crohn's disease revealed by a metagenomic approach. Gut 55:205–211. doi:10.1136/gut.2005.073817. 16188921 PMC1856500

[54] Walters WA, Caporaso JG, Lauber CL, Berg-Lyons D, Fierer N, Knight R. 2011. PrimerProspector: de novo design and taxonomic analysis of barcoded polymerase chain reaction primers. Bioinformatics 27:1159–1161. doi:10.1093/bioinformatics/btr087. 21349862 PMC3072552

[55] Caporaso JG, Lauber CL, Walters WA, Berg-Lyons D, Huntley J, Fierer N, Owens SM, Betley J, Fraser L, Bauer M, Gormley N, Gilbert JA, Smith G, Knight R. 2012. Ultra-high-throughput microbial community analysis on the Illumina HiSeq and MiSeq platforms. ISME J 6:1621–1624. doi:10.1038/ismej.2012.8. 22402401 PMC3400413

[56] Callahan BJ, McMurdie PJ, Rosen MJ, Han AW, Johnson AJ, Holmes SP. 2016. DADA2: high-resolution sample inference from Illumina amplicon data. Nat Methods 13:581–583. doi:10.1038/nmeth.3869. 27214047 PMC4927377

[57] Bolyen E, Rideout JR, Dillon MR, Bokulich NA, Abnet CC, Al-Ghalith GA, Alexander H, Alm EJ, Arumugam M, Asnicar F, Bai Y, Bisanz JE, Bittinger K, Brejnrod A, Brislawn CJ, Brown CT, Callahan BJ, Caraballo-Rodríguez AM, Chase J, Cope EK, Da Silva R, Diener C, Dorrestein PC, Douglas GM, Durall DM, Duvallet C, Edwardson CF, Ernst M, Estaki M, Fouquier J, Gauglitz JM, Gibbons SM, Gibson DL, Gonzalez A, Gorlick K, Guo J, Hillmann B, Holmes S, Holste H, Huttenhower C, Huttley GA, Janssen S, Jarmusch AK, Jiang L, Kaehler BD, Kang KB, Keefe CR, Keim P, Kelley ST, Knights D, et al. 2019. Reproducible, interactive, scalable and extensible microbiome data science using QIIME 2. Nat Biotechnol 37:852–857. doi:10.1038/s41587-019-0209-9. 31341288 PMC7015180

[58] Pruesse E, Quast C, Knittel K, Fuchs BM, Ludwig W, Peplies J, Glöckner FO. 2007. SILVA: a comprehensive online resource for quality checked and aligned ribosomal RNA sequence data compatible with ARB. Nucleic Acids Res 35:7188–7196. doi:10.1093/nar/gkm864. 17947321 PMC2175337

[59] Quast C, Pruesse E, Yilmaz P, Gerken J, Schweer T, Yarza P, Peplies J, Glöckner FO. 2013. The SILVA ribosomal RNA gene database project: improved data processing and web-based tools. Nucleic Acids Res 41:D590–D596. doi:10.1093/nar/gks1219. 23193283 PMC3531112

[60] Knights D, Kuczynski J, Charlson ES, Zaneveld J, Mozer MC, Collman RG, Bushman FD, Knight R, Kelley ST. 2011. Bayesian community-wide culture-independent microbial source tracking. Nat Methods 8:761–763. doi:10.1038/nmeth.1650. 21765408 PMC3791591

[61] Langille MGI, Zaneveld J, Caporaso JG, McDonald D, Knights D, Reyes JA, Clemente JC, Burkepile DE, Vega Thurber RL, Knight R, Beiko RG, Huttenhower C. 2013. Predictive functional profiling of microbial communities using 16S rRNA marker gene sequences. Nat Biotechnol 31:814–821. doi:10.1038/nbt.2676. 23975157 PMC3819121

[62] Kanehisa M, Furumichi M, Tanabe M, Sato Y, Morishima K. 2017. KEGG: new perspectives on genomes, pathways, diseases and drugs. Nucleic Acids Res 45:D353–D361. doi:10.1093/nar/gkw1092. 27899662 PMC5210567

