# Supplementary material for: Microbial Diversity and Composition in Six Different Gastrointestinal Sites among Participants Undergoing Upper Gastrointestinal Endoscopy in Henan, China
Source: Microbiol Spectr. 2022 Apr 25;10(3):e00645-21. doi: 10.1128/spectrum.00645-21 (PMC9241895; doi:10.1128/spectrum.00645-21)
Supplement: SUPPLEMENTAL FILE 1 — Supplemental material. Download spectrum.00645-21-s001.pdf, PDF file, 1.4 MB [file spectrum.00645-21-s001.pdf]

Supplementary Materials

Figure S1 SourceTracker proportion estimates for feces specimens. In SourceTrakcer analysis, the feces specimens were the sink, and the saliva, esophageal swab, cardia biopsy, noncardia biopsy and gastric juice from the same participant were the source.

Figure S2 The LEfSe results of characteristic genera in each GI site. Each bar represented the relative abundance in each specimen.

Table S1 The average relative abundance (%) of the top 15 genera in GI sites. Genera were ordered from high to low according to the relative abundance in saliva. Each cell was the average relative abundance (%) of each genus in different GI sites. For all genera, there were statistical differences among six GI sites. ($P < 0.001$) "0.00" indicated the average relative abundance (%) was not equal to zero but less than 0.01%. "-" indicated the average relative abundance (%) was zero.

Table S2 The characteristic metabolism pathways in different GI sites. [a] Each cell was the logarithmic 2 of mean value in esophagus group, cardia group, noncardia group, gastric juice group and feces group divided by the mean value in saliva group respectively. The q-value of each characteristic pathway was less than 0.05. For each pathway, the log2(Foldchange) of the characteristic group was equal or greater than 5.

Table S3 The *Hp* infection and pH value and the characteristic human disease pathways. [a] Each cell was the logarithmic 2 of mean value in *Hp* positive group divided by the mean value in *Hp* negative group, which was equal or greater than 2. The q-value of each characteristic pathway was less than 0.05. [b] Each cell was the logarithmic 2 of mean value in higher pH group divided by the mean value in lower pH group in gastric juice, which was equal or greater than 2. The q-value of each characteristic pathway was less than 0.05.

Table S4 The relative abundance (%) of the dominant genus in gastric juice. The genera were ordered from high to low based on the relative abundance in HP group. The cell in the *P*-value (ALL) column indicated the P-value of the Kruskal-Wallis test among HP group, H group, P group, LP group, HN group, L group, N group and LN group. "*", "**" and "***" indicated the *P*-value were less than 0.05, 0.01 and 0.001 respectively.

Figure S1 SourceTracker proportion estimates for feces speciemens.

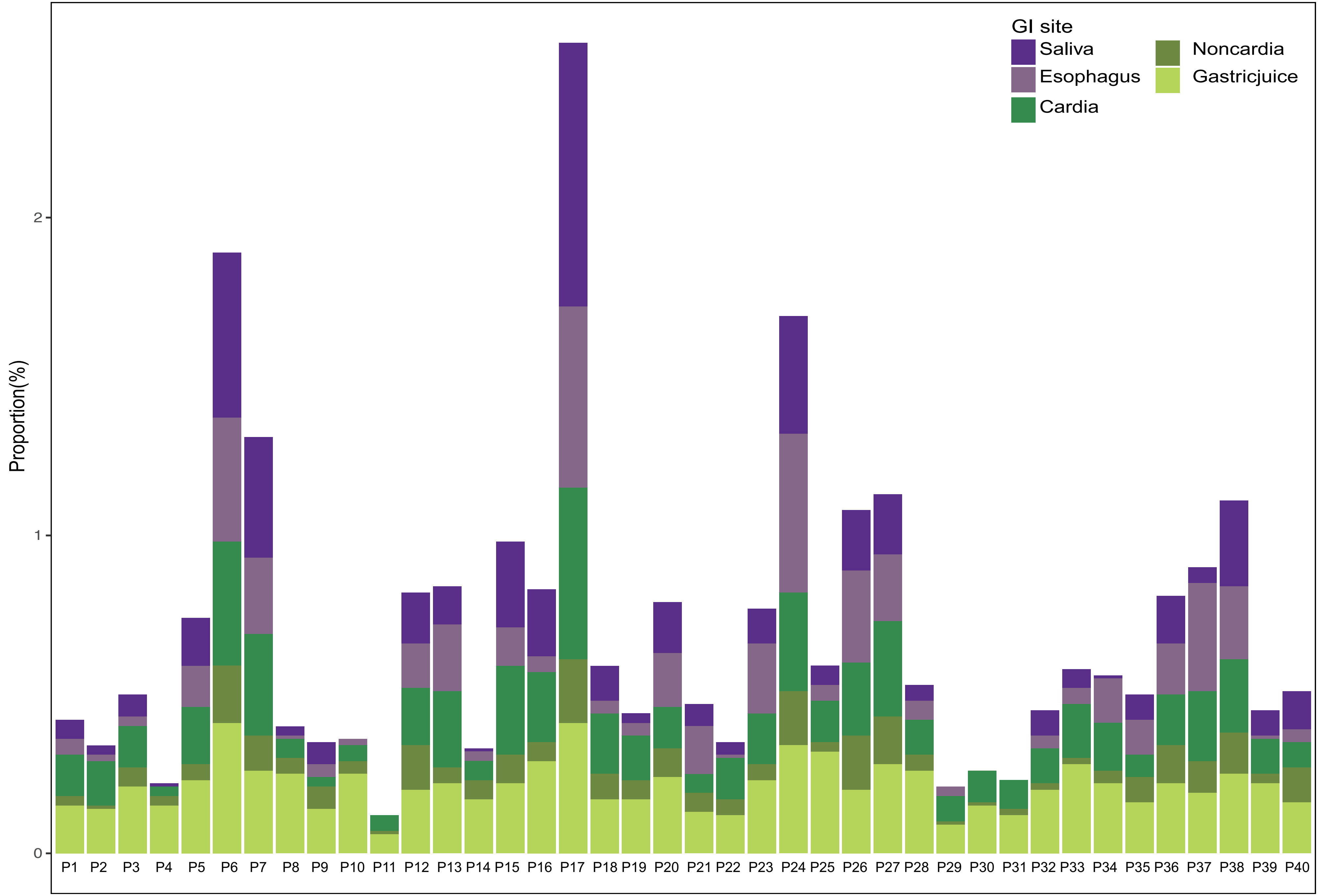

Figure S1 SourceTracker proportion estimates for feces specimens. In SourceTrakcer analysis, the feces specimens were the sink, and the saliva, esophageal swab, cardia biopsy, noncardia biopsy and gastric juice from the same participant were the source.

Figure S2 The LEfSe results of characteristic genera in each GI site.

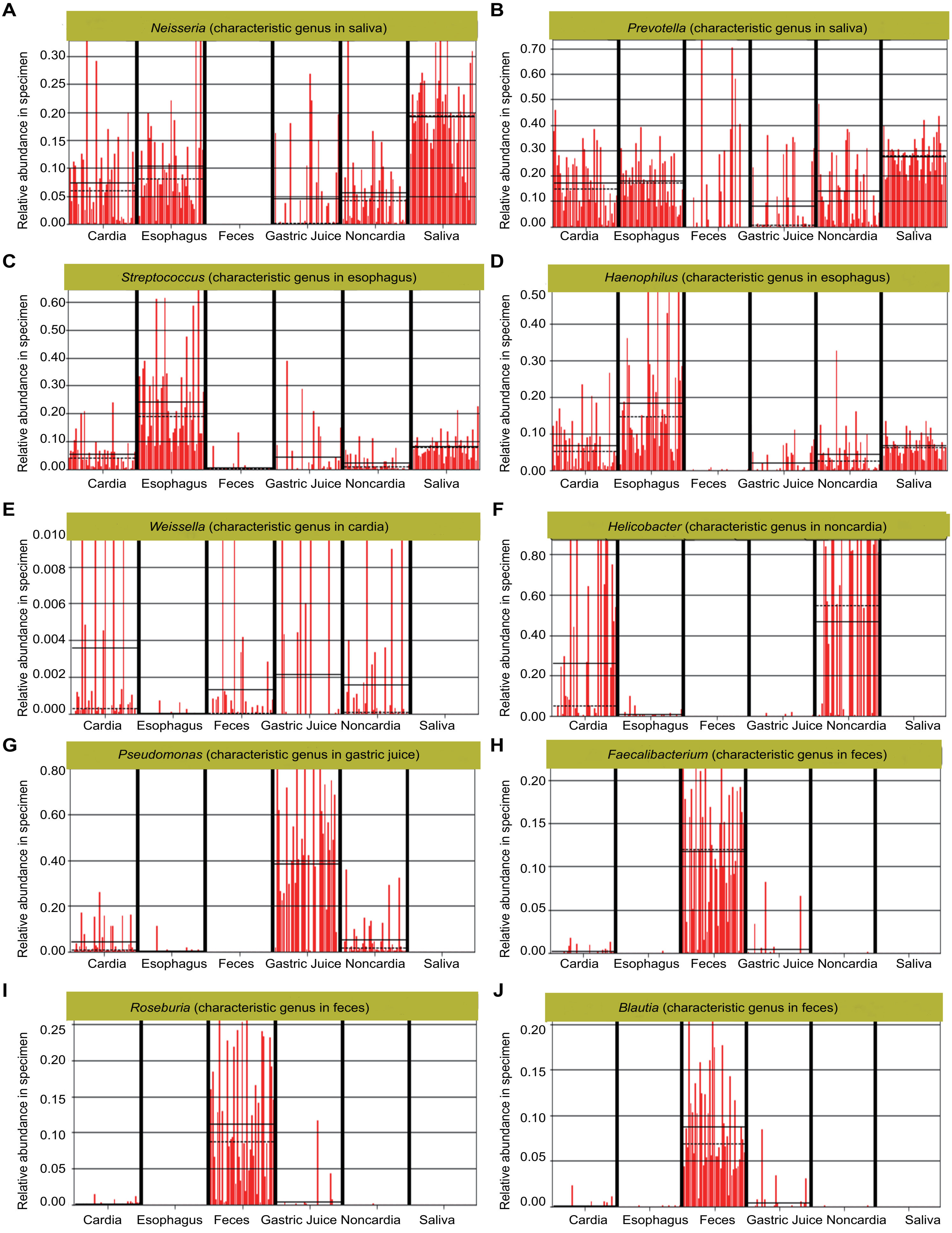

Figure S2 The LEfSe results of characteristic genera in each GI site. Each bar represented the relative abundance in each specimen.
